# Acinar transformed ductal cells exhibit differential mucin expression in a tamoxifen-induced pancreatic ductal adenocarcinoma mouse model

**DOI:** 10.1242/bio.052878

**Published:** 2020-09-07

**Authors:** Kavita Mallya, Dhanya Haridas, Parthasarathy Seshacharyulu, Ramesh Pothuraju, Wade M. Junker, Shiv Ram Krishn, Sakthivel Muniyan, Raghupathy Vengoji, Surinder K. Batra, Satyanarayana Rachagani

**Affiliations:** 1 Department of Biochemistry and Molecular Biology, University of Nebraska Medical Center, Omaha, NE 68198-5870, USA; 2 Sanguine Diagnostics and Therapeutics, Inc., Omaha, NE 68106-1423, USA; 3 Fred and Pamela Buffett Cancer Center, University of Nebraska Medical Center, Omaha, NE 68106, USA; 4 Eppley Institute for Research in Cancer and Allied Diseases, University of Nebraska Medical Center, Omaha, NE 68198-5950, USA

**Keywords:** Mucin, Pancreatic ductal adenocarcinoma (PDAC), Inducible KC (iKC) mouse model

## Abstract

Pancreatic cancer (PC) is acquired postnatally; to mimic this scenario, we developed an inducible Kras^G12D^; Ptf1a-CreER™ (iKC) mouse model, in which Kras is activated postnatally at week 16 upon tamoxifen (TAM) administration. Upon TAM treatment, iKC mice develop pancreatic intraepithelial neoplasia (PanIN) lesions and PC with metastasis at the fourth and fortieth weeks, respectively, and exhibited acinar-to-ductal metaplasia (ADM) and transdifferentiation. Kras activation upregulated the transcription factors Ncoa3, p-cJun and FoxM1, which in turn upregulated expression of transmembrane mucins (Muc1, Muc4 and Muc16) and secretory mucin (Muc5Ac). Interestingly, knockdown of Kras^G12D^ in multiple PC cell lines resulted in downregulation of MUC1, MUC4, MUC5AC and MUC16. In addition, iKC mice exhibited ADM and transdifferentiation. Our results show that the iKC mouse more closely mimics human PC development and can be used to investigate pancreatic ductal adenocarcinoma (PDAC) biomarkers, early onset of PDAC, and ADM. The iKC model can also be used for preclinical strategies such as targeting mucin axis alone or in combination with neo-adjuvant, immunotherapeutic approaches and to monitor chemotherapy response.

## Abstract (precis)

**Summary:** In this study, we developed a tamoxifen-inducible transgenic mouse model that closely mimics the age-related onset of pancreatic cancer (PC). We showed that activation of *Kras* (iKC) specifically regulates transcription factors that promote mucin expression and acinar to ductal metaplasia, whereas, loss of *Kras^G12D^* allele in PC cells resulted in the reduction of mucin proteins such as MUC1, MUC4, MUC5AC and MUC16.

## INTRODUCTION

Pancreatic ductal adenocarcinoma (PDAC) is a lethal malignancy with an extremely poor prognosis, having a 5-year survival rate of less than 9% (Siegel et al., 2020). The lethality of PDAC is due to late diagnosis, early local invasion, high metastatic potential and resistance to current chemo-radiotherapies (Bailey et al., 2014; Chakraborty et al., 2011). The major risk factors for PDAC initiation and progression include genetic mutations in *KRAS*, *INK4A* (p16), *Trp53*, *DPC4* and *BRCA2* as well as other risk factors like cigarette smoking, obesity and diabetes mellitus (Guerra et al., 2007; Habbe et al., 2008; Hingorani et al., 2003; Jones et al., 2008). *Kirsten rat sarcoma viral oncogene homolog* (*Kras*) is a major driver in PDAC development and the most frequently mutated gene, with constitutive activation in 70–90% of PDAC patients (Bailey et al., 2014; Feldmann et al., 2007; Jones et al., 2008; Prior et al., 2012). Further, accumulation of genetic mutations in other tumor pathway genes ultimately leads to invasive PDAC (Khan et al., 2017).

Early detection of PDAC is difficult because most PDAC patients (>95%) are diagnosed with advanced disease. Since pre-neoplastic human samples are rarely available, genetically engineered mouse models (GEMM) can be used to identify and analyze molecules specifically involved in the initiation, progression and advancement of the disease. Since 2003, the *Kras^G12D^;Pdx1-Cre* (KC) and *Kras^G12D^;Trp53^R172H^;Pdx1-Cre* (KPC) mouse models (Hingorani et al., 2003; Hingorani et al., 2005; Rachagani et al., 2012 b) have been used extensively to study the pathogenesis of pancreatic cancer (PC). The tumors and the spectrum of pancreatic intraepithelial neoplasia (PanIN) lesions express a variety of molecules associated with PDAC initiation and progression, including Muc1, Muc4, Muc5Ac, Muc16, Her-1, Her-3, PD-2/hPaf-1, Ncoa3, Mmp-9 and Cox-2 (Dey et al., 2014; Hingorani et al., 2003; Lakshmanan et al., 2015; Rachagani et al., 2012b). However, in these models, the *Kras* oncogene activation/*Trp53* inactivation occurs during prenatal life, throughout all pancreatic cell types; in contrast, PDAC is an age-related disease with *Kras* activation occurring late in adult life.

In humans, the incidence of PDAC increases sharply after age 50, with most diagnoses at an advanced age (65–85 years), and the earliest reported cases at approximately age 30 (Higuera et al., 2016). The risk of men and women developing PDAC is about 100 and 87 times higher, respectively (Chakraborty et al., 2011; Higuera et al., 2016). This age-related onset is also observed in experimental rodents as the incidence of spontaneous tumors increases after 300 days of age (Anisimov et al., 2005). However, in KC and KPC mouse models, Cre recombinase is induced at embryonic day 8.5, which leads to activation of *Kras* or inactivation of *Trp53* (Dey et al., 2014; Hingorani et al., 2003; Hingorani et al., 2005; Rachagani et al., 2012 b). PanIN lesions develop as early as week 10 in KC and week 5 in KPC models, with tumors and metastasis at 50 and 25 weeks of age, respectively (Hingorani et al., 2003; Hingorani et al., 2005; Rachagani et al., 2012 b; Torres et al., 2013). This early tumor onset may be a possible reason why therapeutics that are successful in preclinical studies fail in clinical trials. In addition, previous studies have shown that PanIN lesions can arise from pancreatic acinar cells through a reprogramming process called acinar-to-ductal metaplasia (ADM) (De La et al., 2008; Feldmann et al., 2007; Habbe et al., 2008), thus serving as a reservoir for PDAC development.

Genes encoding transcription factors critical in pancreatic progenitor cell development have been harnessed to drive Cre recombinase expression in specific progenitor cell populations to conditionally activate gene expression (Magnuson and Osipovich, 2013). These include the well-characterized homeodomain-containing transcription factor, pancreas and duodenum homeobox 1 (Pdx1), and pancreas transcription factor 1a (Ptf1a), which drive formation of endocrine and exocrine pancreatic cell lineages. Lineage tracing with Pdx1-Cre or Ptf1a-Cre (Kawaguchi et al., 2002) Cre-driver lines marks all pancreatic cell types, as both of these transcription factors are expressed in pancreatic multipotent cells (PMC) (Magnuson and Osipovich, 2013). However, since Ptf1a transcription factor expression is retained only by differentiated acinar cells (following embryonic day 8.5–9.5), we acquired a Cre-driver line that allowed us to (1) specifically promote CRE recombination within the acinar cell population and (2) facilitate temporal control of recombination/*KRAS* expression in postnatal life using Cre^ER^ (Feil et al., 1996). This would not be possible with Pdx1, which functions both in early pancreas PMC development and later in mature islet endocrine cells.

*Kras* is the critical driver of several cancers, including PC and it is the most frequently mutated gene (>79%) in PC patients (Haas et al., 2017; Rachagani et al., 2011). Interestingly, mucins were shown to be predominantly expressed in *Kras*-dependent tumors (Rachagani et al., 2012b). Previously, we showed that genetic ablation of the *Kras-*specific G12D allele in the MUC4-overexpressing PC cell line (CD18/HPAF) lead to a significant reduction in proliferation, migration, anchorage-dependent growth and ability to form metastasis in mice (Rachagani et al., 2011). Furthermore, a gel-forming mucin, Muc5Ac, was demonstrated to be a critical determinant of poor survival in *Kras*-mutated lung cancer patients (Bauer et al., 2018). Another membrane-bound mucin, MUC16, is aberrantly overexpressed and is involved in the pathobiological landscapes of pancreatic and lung adenocarcinoma (Haridas et al., 2011; Lakshmanan et al., 2017). Currently, there are no clinically tested *Kras* inhibitors. Hence, drugs that indirectly affect activated *Kras* activity through mucins might bring a breakthrough in *Kras*-dependent tumor treatment. In this context, our inducible model could be used for testing small molecule inhibitors that affect mucins; thereby, indirectly targeting the ‘undruggable’ target, *Kras*.

In our study design, we generated an inducible KC mouse model, by intercrossing *LSL-Kras^G12D^ mice* (knockin allele for mutant *Kras^G12D^*) (Jackson et al., 2001) with Ptf1a-CreER™ mice (knockin allele for Ptf1a-CreER™) (Kopinke et al., 2012) that have abolished *Ptf1a* gene function and express the CreER^TM^-fusion protein from the *Ptf1a* promoter/enhancer elements. Upon tamoxifen treatment, the PTF1a-CRE^ER^-fusion protein moves from the cytoplasm to the nucleus. Cre-mediated recombination results in deletion of the floxed stop sequence Lox-Stop-Lox [(LSL) cassette] and activation of *Kras^G12D^* in pancreatic acinar cells. Thus, tamoxifen administration in the compound strain, *Kras^G12D^;Ptf1a-CreER*™ (iKC), postnatally, will specifically activate *Kras^G12D^* only in the acinar compartment, i.e. after acinar cell terminal differentiation and not in ductal and endocrine cells. We examined the pancreas-specific expression of mucin proteins (Muc1, Muc4, Muc5Ac, and Muc16) and the transcriptions factors (Ncoa3, p-cJun, and FoxM1) involved in their expression. We also investigated if activation of *Kras^G12D^* in the acinar compartment could drive reprogramming of acinar cells to express the ductal cell marker cytokeratin-19 (CK19).

## RESULTS

### PDAC progression in the tamoxifen-induced iKC mouse model

**Fig. 1.**
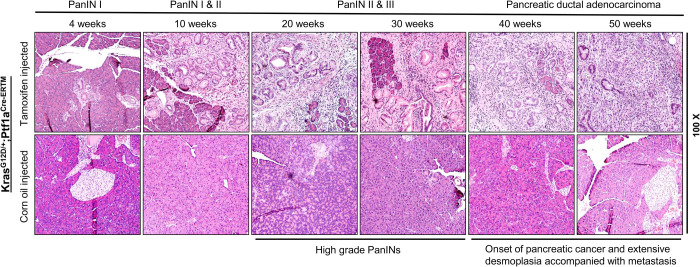
**PC progression in the tamoxifen-induced iKC mouse model**. Pancreatic tissues were isolated from corn-oil- and tamoxifen-treated iKC mice at 4, 10, 20, 30 and 50 weeks of age. The tissues were paraffin-embedded and formalin-fixed. Sections (4 µm) were stained with H&E. Pre-cancerous lesions (PanINs) were not detected at any point in corn-oil-treated animals. By 4 weeks after tamoxifen injection, PanINs had formed; they progressed to high-grade lesions by 30 weeks and PC by 50 weeks.

To specifically express *Kras^G12D^* in the adult acinar cell lineage, we crossed LSL *Kras^G12D^* mice with *Ptf1a-CreER*™ mice and generated the iKC inducible mouse model. In this model, tamoxifen is used to activate Cre, which induces mutant *Kras^G12D^* expression in acinar cells of 16-week-old animals, while littermate controls are injected with corn oil. Mice were euthanized at 4, 10, 20, 30, 40, and 50 weeks (*n*=4 for each time point) post-treatment. Time to onset of tumor initiation, progression (degree of disease), and the impact of *Kras^G12D^* activation on mucin expression were determined. During necropsy, tumors were excised, and distant organs were examined for metastatic lesions. In tamoxifen-treated animals, we observed both micro- and gross metastases to regional lymph nodes, liver, spleen, diaphragm, and mesenteric lymph nodes (data not shown). Pancreata from tamoxifen- and corn-oil-treated mice at each time point were stained with Hematoxylin and Eosin (H&E) to assess differences in histopathology between the groups. Mice treated with tamoxifen displayed pre-neoplastic PanIN I lesions by 4 weeks post-tamoxifen treatment (Fig. 1, upper panel). By 10 weeks, low-grade PanIN lesions (PanIN I and II) were observed, and high-grade (PanIN III) lesions were prevalent at 30 weeks after tamoxifen injection (Fig. 1, upper panel). Pancreatic (PDAC) tumors and extensive desmoplasia were observed at 40–50 weeks post-tamoxifen treatment (Fig. 1, upper panel) and were accompanied with metastasis. Corn-oil-treated mice did not display any histological changes and exhibited normal pancreas histology (Fig. 1, lower panel). Thus, the iKC model closely resembles the clinical scenario of PanIN formation and PDAC development.

### Expression of acinar marker amylase and ductal marker cytokeratin-19 in the iKC mouse model

**Fig. 2.**
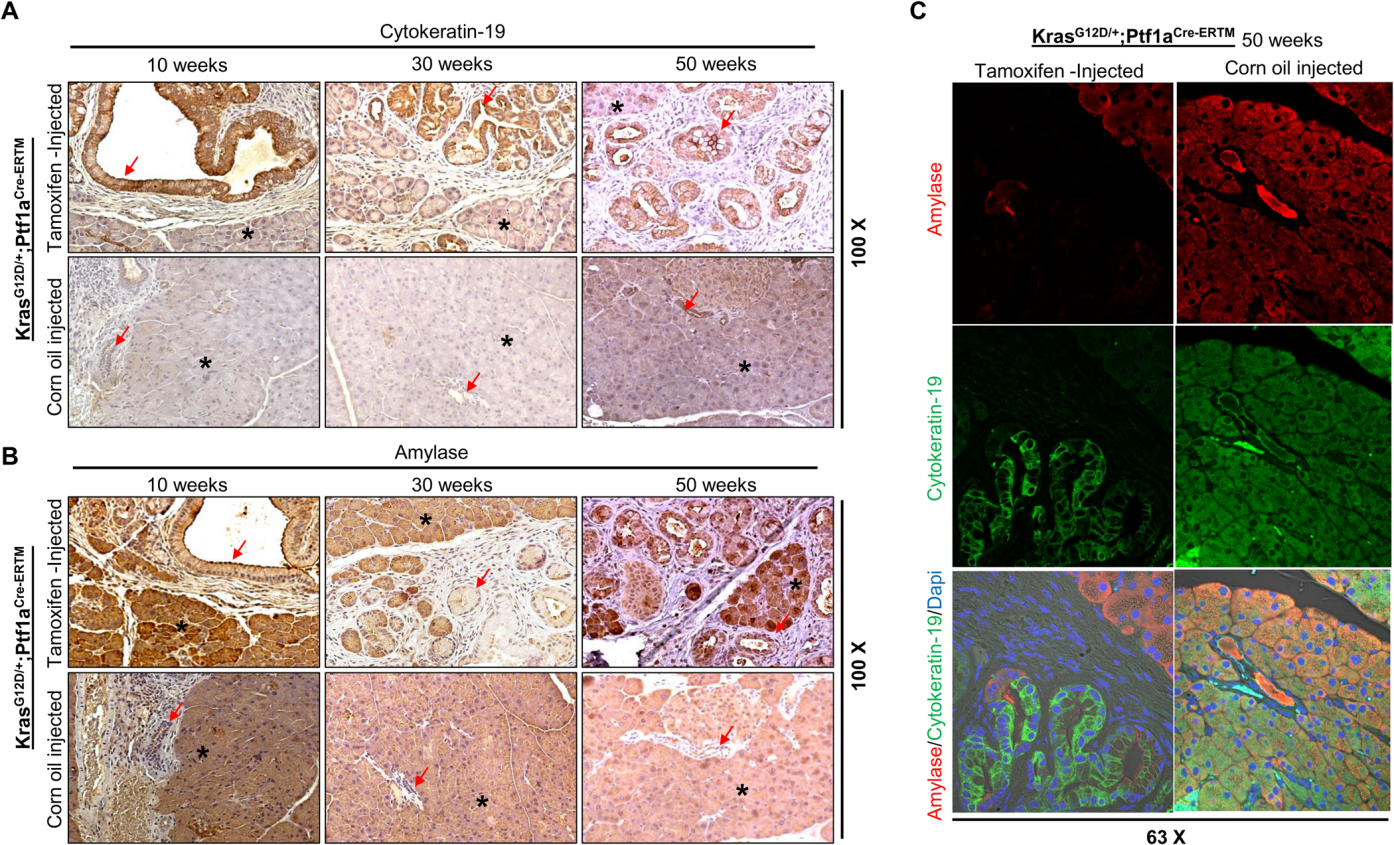
**Expression of acinar marker amylase and ductal marker cytokeratin-19 in iKC mouse model.** IHC studies using CK19 and amylase antibodies were performed on pancreatic tissues isolated from both corn-oil- and tamoxifen-treated iKC mice. (A) Expression of CK19 was not detected at any time point in the corn-oil-treated mouse tissues (lower row). In contrast significant upregulation of CK19 was detected in tamoxifen-induced mouse tissues (upper row). (B) The expression of amylase was observed in the acinar compartment of the pancreas (upper and lower panels) and the transdifferentiated ductal compartment (upper panel). (C) Immunofluorescence confocal microscopy revealed that amylase and CK19 were coexpressed in ducts undergoing transdifferentiation in the tamoxifen-induced mice.

In our previous study (Dey et al., 2014), we reported that PanIN lesions/PDAC can arise from a unique intermediate ADM-like cellular transition; this was shown by localization of epithelial/ductal cell marker, CK-19; acinar cell marker, amylase; and PD2/Paf1 (pancreatic differentiation 2) in KC and cerulin-injected KC mouse models. We showed that knockdown of PD2/Paf1 leads to an increase in the epithelial cell/ductal marker CK-19 and a decrease in acinar cell marker amylase in pancreatic acinar cells (Dey et al., 2014). Thus, we wanted to confirm that the iKC model also exhibits this acinar-to-ductal transdifferentiation process. We performed immunohistochemistry (IHC) and immunolocalization assays in serial sections of pancreas obtained from tamoxifen- and corn-oil-administered (10, 30, and 50 weeks of treatment) mice. As expected, CK-19 expression was observed in ductal epithelium arising from acinar cells undergoing acinar to ductal metaplasia in tamoxifen-treated mice (Fig. 2A upper panel) and was absent in corn-oil-treated mice (Fig. 2A lower panel). Similarly, acinar cells specifically expressed amylase (Fig. 2B upper panel) in the corn-oil-treated and not in tamoxifen-treated mouse pancreas (Fig. 2B lower panel). Next, we examined whether the intermediate acinar-to-ductal-like cells of the newly developing pancreatic ducts co-expressed CK-19 and amylase in tamoxifen-injected mice. CK-19 and amylase frequently co-localized to newly forming ducts that were undergoing the transformation process (Fig. 2C). These results suggest that tamoxifen induces *Kras* specifically in the acinar compartment of the iKC mouse, and this model will be useful for exploring gene signatures involved in ADM.

### Expression of transmembrane mucins Muc1 and Muc4 in the iKC mouse model

**Fig. 3.**
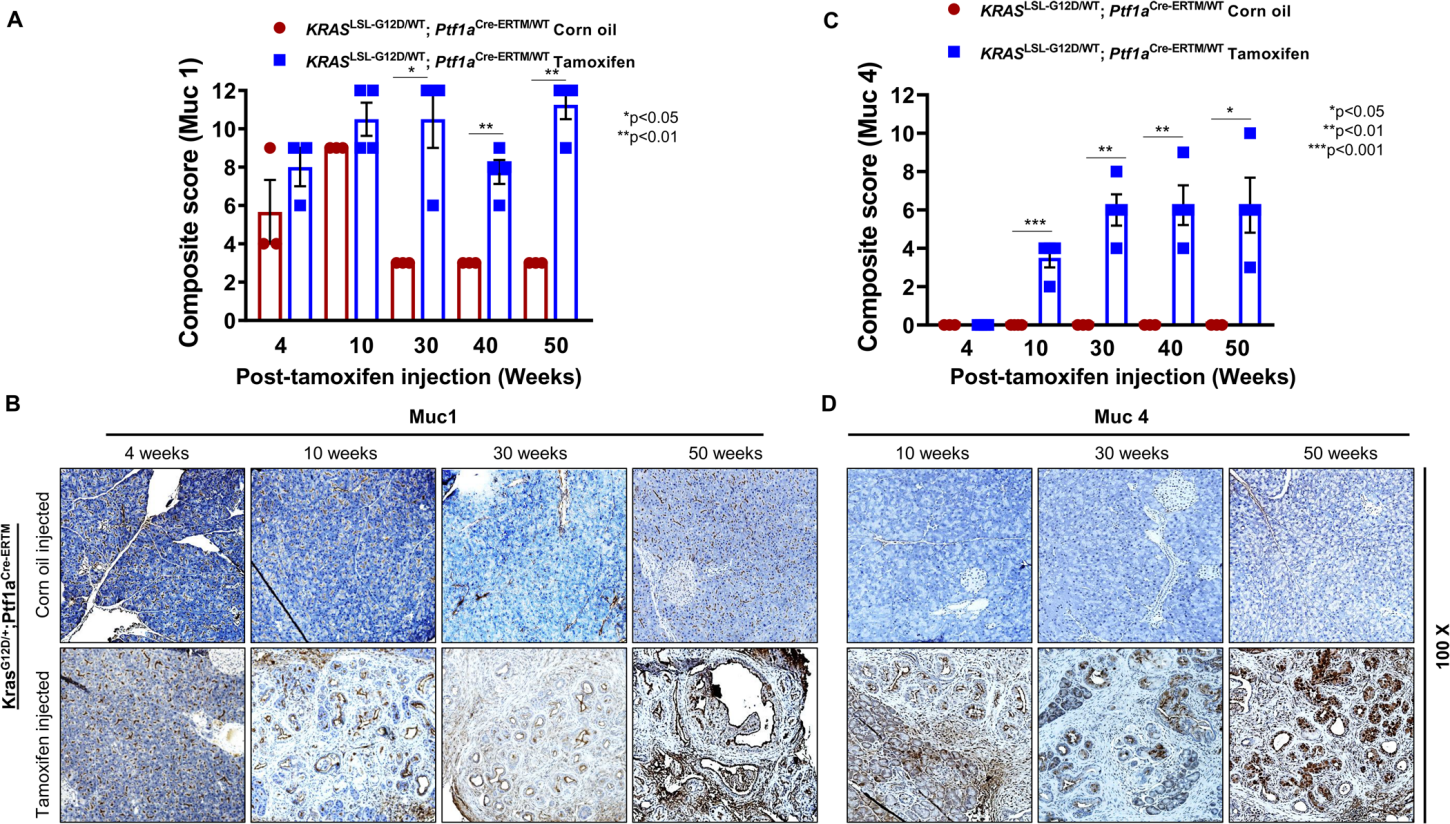
**Expression of transmembrane mucins Muc1 and Muc4 in the iKC mouse model.** IHC studies were performed to analyze Muc1 and Muc4 protein expression during the progression of PC in the iKC model. (A) Composite IHC scores for Muc1 show a significant increase from 10 to 50 weeks after tamoxifen injection. (B) Muc1 was expressed in iKC mice treated with corn oil or tamoxifen but was higher in tamoxifen-treated mice compared to the control mice at each time point. (C) Composite IHC scores for Muc4 show a significant increase from 10 to 50 weeks after tamoxifen injection, with no expression in corn-oil-treated mice. (D) Muc4 expression was not detected in pancreatic tissues obtained from corn-oil-treated iKC mice (upper row) or iKC mice treated with tamoxifen for only 4 weeks. Muc4 expression was detected in iKC pancreatic tissues at 10, 20, 30 and 50 weeks after tamoxifen treatment (bottom row). Muc4 expression significantly increased with cancer progression.

Muc1 is involved in the progression of various cancers, including PC (Andrianifahanana et al., 2001; Bafna et al., 2010; Kaur et al., 2013; Tsutsumida et al., 2006; Westgaard et al., 2009). In our earlier study using the KC mouse model, we showed Muc1 expression is associated with the progression of mouse PDAC (Rachagani et al., 2012b). Therefore, we investigated Muc1 expression in the iKC model. Our results reveal that Muc1 expression increased progressively in the pancreas of tamoxifen-treated iKC mice, starting from 4 weeks to 50 weeks. Average composite IHC scores ranged from 7.5 to 11.25, showing a significant difference from littermate control animals at 30 (*P*<0.05), 40 (*P*<0.01) and 50 (*P*<0.01) weeks post-treatment (Fig. 3A). Muc1 expression was predominantly observed in the epithelial layer of the pancreatic ducts. In contrast, a basal level of Muc1 expression was observed in the pancreas of corn-oil-treated animals and did not increase between 30, 40 and 50 weeks of treatment (Fig. 3B). Muc1 expression in the iKC model reflects what was previously reported in the study of the KC mouse model (Rachagani et al., 2012b).

MUC4 is highly upregulated in pancreatic and ovarian cancers and is associated with poor prognosis (Chaturvedi et al., 2007; Ponnusamy et al., 2010; Rachagani et al., 2012a). Studies from our lab have demonstrated MUC4 is not expressed in the normal pancreas and that MUC4 *de novo* expression in human PDAC (Andrianifahanana et al., 2001) plays a vital role in the progression and advancement of PC (Bafna et al., 2010; Chaturvedi et al., 2007; Kaur et al., 2013; Lakshmanan et al., 2015; Moniaux et al., 2004; Rachagani et al., 2012a). Study of Muc4 expression in KC mice showed Muc4 RNA and protein are specifically expressed in the ductal epithelial cells of the pancreas (Rachagani et al., 2012b). The expression of Muc4 in the iKC model mirrored this expression pattern. IHC results showed Muc4 is localized to the cell membrane and cytoplasm in the majority of the pancreatic ductal epithelial cells (Fig. 3D lower panel). Muc4 progressively increased with its average IHC composite score increasing from 3.5 at 10 weeks (*P*<0.001) to 7.33 at 50 weeks (*P*<0.05) post-treatment (Fig. 3C). This was in sharp contrast to no Muc4 detected in the pancreas of corn-oil-treated littermate control mice of all age groups (10–50 weeks) (Fig. 3D upper panel).

### Expression of secreted Muc5Ac in the iKC mouse model

**Fig. 4.**
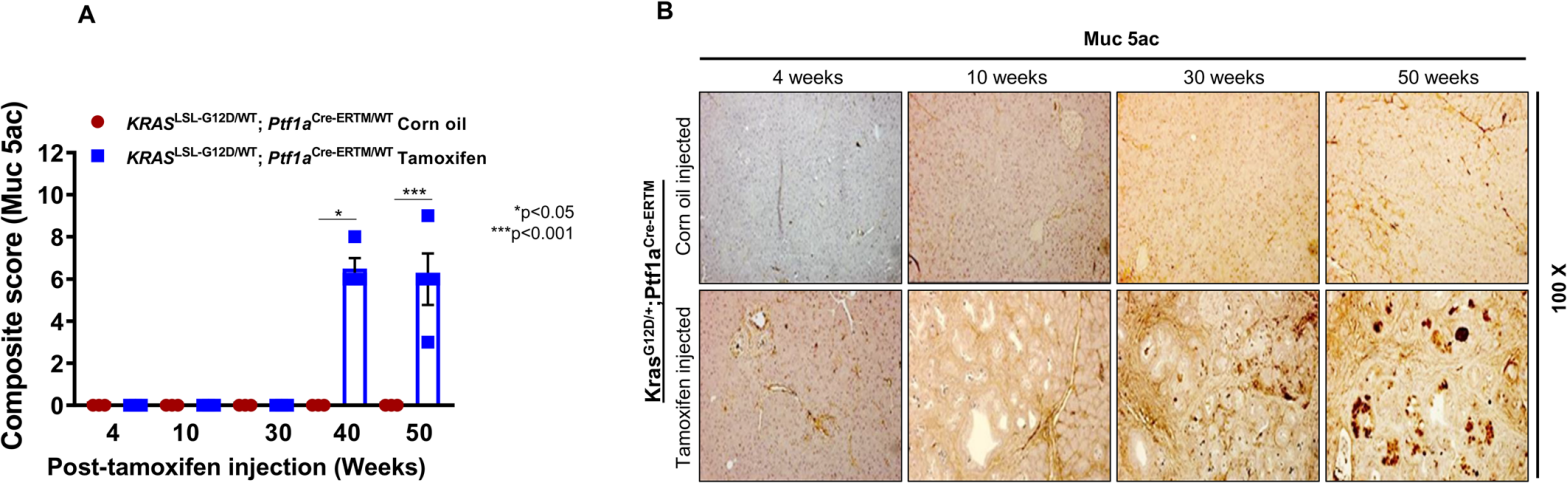
**Expression of secreted Muc5Ac in the iKC mouse model.** Immunohistochemistry was performed to assess Muc5Ac protein expression during the progression of PC in the iKC model. (A) Composite IHC scores for Muc5Ac expression in tamoxifen- and corn-oil-treated iKC mice from 4 to 50 weeks of age. (B) Muc5Ac was detected in the pancreas of tamoxifen-treated mice only at 40 and 50 weeks after tamoxifen injection and not at 4, 10 and 30 weeks. Pancreata isolated from control animals were negative for Muc5Ac expression.

MUC5AC is overexpressed in human PDAC but is undetectable in the normal pancreas (Kaur et al., 2017). These findings are similar to Muc5Ac expression (Bafna et al., 2010; Chaturvedi et al., 2007; Kaur et al., 2013; Rachagani et al., 2012a,b) in the KC model, where Muc5Ac expression is first observed in 10-week-old animals. However, *de novo* expression of Muc5Ac occurred much later in the iKC model, at 40 weeks of post-tamoxifen treatment (postnatal 56-week-old animals). The Muc5Ac average IHC composite score increased from 6.5 at 40 weeks (*P*<0.05) to 7.0 at 50 weeks (*P*<0.001) post-treatment **(**Fig. 4A and B, lower panel). As observed in KC mice, *de novo* expression increased progressively in the iKC model. This was in sharp contrast to all corn-oil-treated control groups, where there was no expression of Muc5Ac was observed in the pancreas (Fig. 4B, upper panel).

### Expression of Muc16 in the iKC mouse model

Previously, we reported that MUC16 is overexpressed in pancreatic, breast and lung cancer (Haridas et al., 2011; Lakshmanan et al., 2012; Muniyan et al., 2016) and promotes a rapid G2/M checkpoint transition through its interaction with JAK2/STAT3, leading to accelerated proliferation. Further, MUC16 also inhibits apoptosis by suppressing TRAIL-mediated signaling (Lakshmanan et al., 2012). In another study, we showed that the MUC16 C-terminal region (283 amino acids of C-terminus) was responsible for increasing the tumorigenic and metastatic potential of PC cells by promoting the JAK2/STAT3 pathway, that results in upregulation of *LMO2* and *NANOG* genes (Das et al., 2015). Recently, we found that MUC16 can interface between extracellular and intracellular proteins, facilitating PC metastasis (Muniyan et al., 2016). Hence, to build on our previous studies of *MUC16* in PC and other cancers, we examined the expression status of Muc16 in the iKC model. Our IHC analysis revealed that *Muc16* expression increases progressively from 10 to 50 weeks in tamoxifen-treated mice (Fig. S2, right column). No reactivity was observed in the corn-oil-treated mouse pancreas from 4 to 50 weeks of age (Fig. S2, left column), which may indicate weak immunoreactivity of the antibody in normal pancreas (Wang et al., 2008).

### Depletion of mutated Kras decreases mucin expression in human PC cell lines

**Fig. 5.**
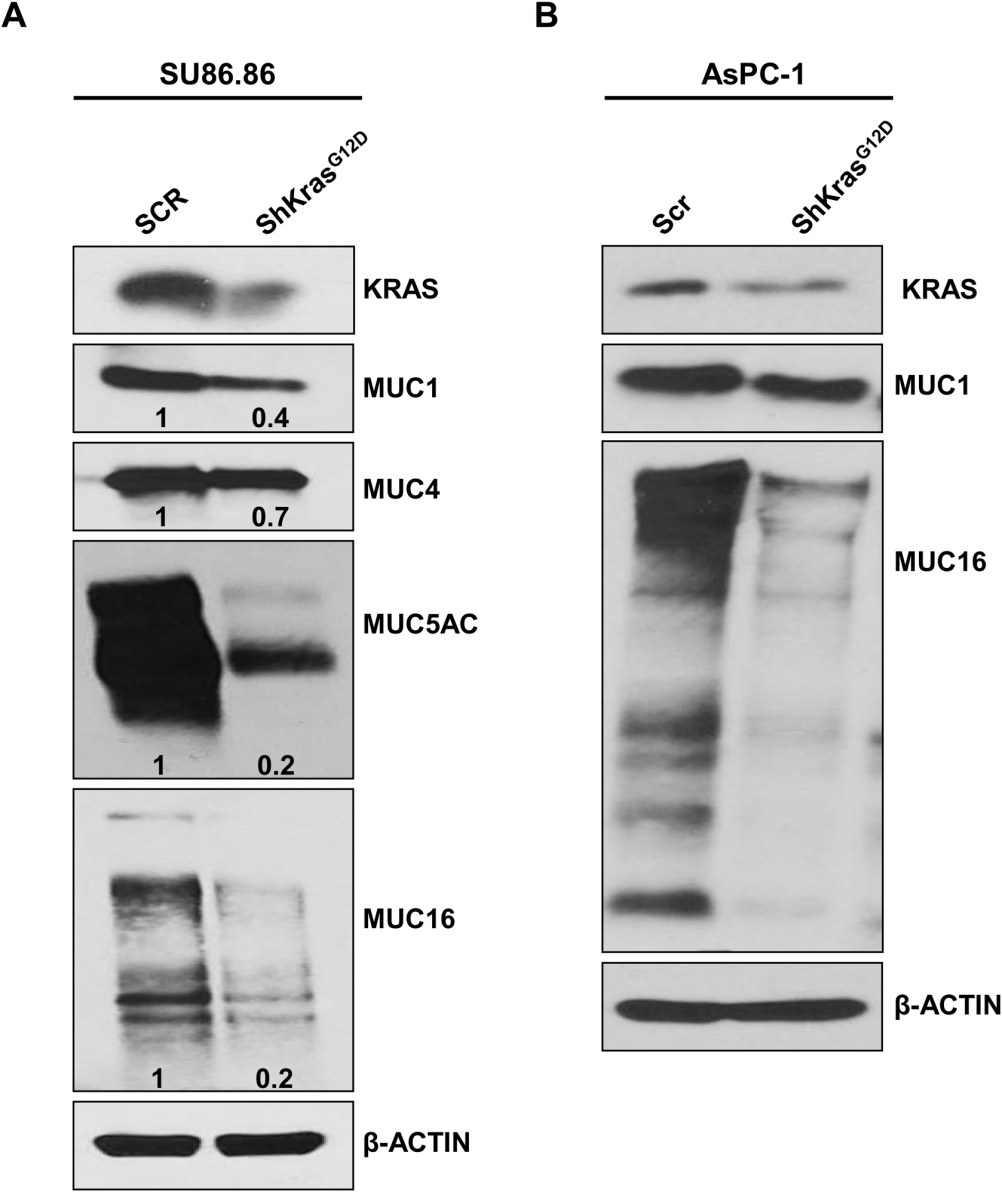
**Depletion of mutated *Kras*** **decreases mucin expression in human PC cell lines.** (A) Transient knockdown of *Kras* was performed in Su86.86 cells. Western blot analysis using anti-MUC1, -MUC4, -MUC5AC and -MUC16 antibodies showed downregulation of mucin proteins MUC1, MUC4, MUC5AC and MUC16 upon *Kras* knockdown. (B) Stable knockdown of *Kras* in AsPC-1 cells using the pRetro.Puro vector carrying an shRNA against mutated *Kras* led to significant downregulation of MUC1 and MUC16 in AsPC-1 cells. β-actin served as the internal loading control.

In a previous study, we showed that mosaic *Cre* activation driven by the Pdx1 promoter results in *Kras* activation throughout all pancreatic cell types and aberrant mucin expression in the KC mouse model of PDAC (Rachagani et al., 2012b). However, we were uncertain how specific targeting of *Kras* activation to the pancreatic acinar cell population would impact mucin expression. In addition to observing the resulting increase in mucin expression in iKC mice, we also investigated transient knockdown of mutated *Kras^G12D^* in human PC cell lines using shRNA/siRNA, and observed significantly decreased MUC1, MUC4, MUC5AC MUC16 mRNA and protein expression. Our results suggest that mutated *Kras* either directly or indirectly modulates (increases) mucin expression in human PC cells (Fig. 5A,B; Fig. S3).

### *Kras* regulates mucins via upregulation of transcription factors

**Fig. 6.**
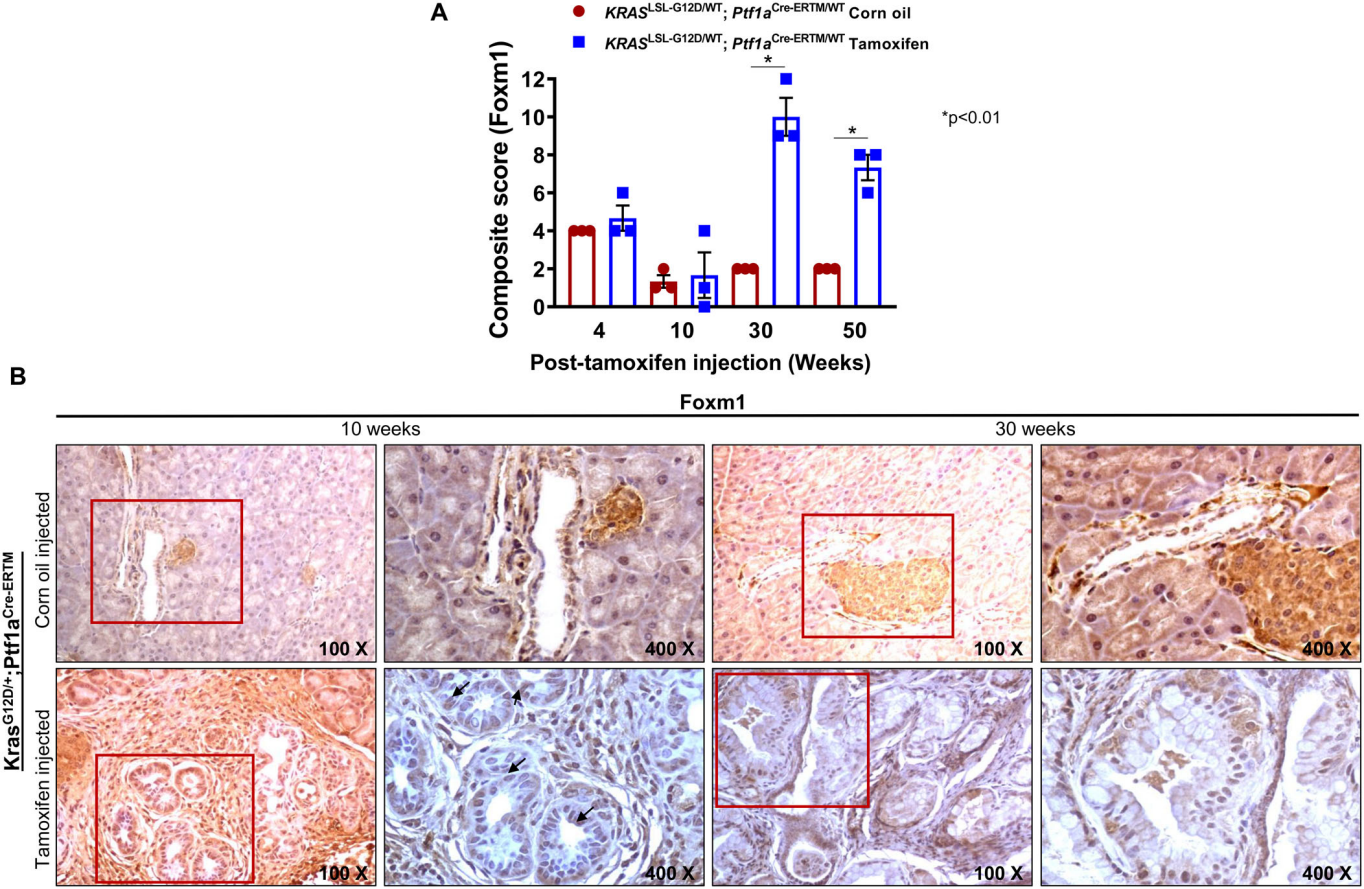
**Expression of FoxM1 in iKC mice after 10 and 30 weeks of tamoxifen treatment.** IHC analysis using FoxM1 antibody on pancreatic tissues isolated from corn-oil- and tamoxifen-treated iKC mice. (A) Bar graph showing quantification of immunohistochemical evaluation of FOXM1 expression in pancreas excised from corn-oil- and tamoxifen-treated mice. (B) The expression of FoxM1 in the nucleus was low in the corn-oil group (basal level), but it increased significantly upon tamoxifen injection.

To investigate how mutant *Kras* regulates mucin expression, we investigated the expression profile of three transcription factors that have been previously shown by us (Kumar et al., 2015) and other groups (Jonckheere and Van Seuningen, 2018) to be associated with mucin regulation in PDAC (Kong et al., 2013; Kumar et al., 2015; Ponnusamy et al., 2010; Rachagani et al., 2012a). Serial sections from the iKC progression model were immunostained for Ncoa3, p-cJun and FoxM1 transcription factors. IHC was performed on pancreatic tissues from iKC mice to confirm that the strong association of NCOA3 with MUC4 and MUC1 expression in human- and mouse- (KC model) PDAC tissues. In iKC PC tissues that were positive for both Muc1 and Muc4, we observed that Ncoa3 was predominantly expressed in the nuclei of pancreatic ductal cells from 10 to 50 weeks and was associated with higher intensity of Muc1- and Muc4-protein expression (increased with Muc1 and Muc4 expression). In contrast, the pancreatic ducts of 10- to 50-week-treated control mice were Ncoa3 negative (Fig. S4A). We also observed upregulation of p-cJun expression in the nucleus of pancreatic ductal cells of iKC mice from 10 to 50 weeks after tamoxifen injection, with no expression of p-cJun detected in the pancreas of control mice (Fig. S4B). A similar upregulation of FoxM1 was observed in mice 30 (*P*<0.01) to 50 (*P*<0.01) weeks of age, treated with tamoxifen as compared to control mice (Fig. 6A,B). Overall, our results suggest that the inducible activation of *Kras* upregulates transcription factors Ncoa3, pcJun and FoxM1, which, in turn, upregulate mucin expression in the pancreas, reflecting our findings that were first reported in the KC model.

## DISCUSSION

For the last two decades, PC researchers have debated the pancreatic cell type from which PDAC originates. Several studies were performed to address this mystery but were inconclusive because human pre-neoplastic tissues were not available (Bailey et al., 2014; Feldmann et al., 2007; Habbe et al., 2008; Higuera et al., 2016) and studies in model systems were not convincing. Our previously published KC mouse model allowed us to capture and study the spectrum of PanIN I-III lesions that progress to PDAC (Hingorani et al., 2003; Rachagani et al., 2012b) with a longer latency than the KPC model. However, certain limitations have been reported in the KC and KPC models such as an ‘extra’-pancreatic phenotype (due to leaky expression of the *Pdx1-Cre* allele in other tissues such as in the skin, the oral mucosa, the perianal, gastric and adrenal regions) (Hingorani et al., 2003, 2005; Rachagani et al., 2012b). Further, PDAC is an age-related disease that shows a steep increase in incidence after 50 years of age (Chakraborty et al., 2011; Higuera et al., 2016). Similarly, the incidence of spontaneous tumors steadily increases in experimental rodents older than 300 days (Anisimov et al., 2005). Yet, the widely used KC and KPC mouse models activate CRE during embryonic development and animals present with PDAC early in postnatal life (Hingorani et al., 2003, 2005; Rachagani et al., 2012b; Torres et al., 2013). To minimize these concerns, we introduced *Cre* expression under the control of an alternative promoter (Ptf1a-CreER™), which has allowed us to induce *Kras* activation postnatally at 16 weeks of life, specifically in acinar cells. This design enabled our study of PanIN progression from the acinar cell lineage and confirmed findings previously made in the KC model under the control of the Pdx1 promoter. The iKC mice developed low-grade PanIN lesions by 4 weeks of treatment, with PDAC and metastasis by 40 weeks post-tamoxifen induction.

A previous study from our lab demonstrated an acinar-to-ductal cell transdifferntiation process in the KC model, using acinar-specific amylase and ductal specific CK-19 as lineage markers (Dey et al., 2014). We assessed these markers using the newly developed iKC model and confirmed this finding. As expected, amylase and CK19 colocalized in metaplastic ducts that were undergoing transdifferentiation following *Kras* activation. Thus, like the KC model, the iKC model can be appropriately used to explore genes related to the ADM transdifferentiation process (Dey et al., 2014; Rachagani et al., 2012b).

We next evaluated the expression of mucin proteins that are known to have direct roles in PC pathogenesis (Andrianifahanana et al., 2001; Bafna et al., 2010; Kaur et al., 2013). An earlier study showed that Muc1, Muc4 and Muc5Ac were overexpressed during progression of PDAC in the KC model (Rachagani et al., 2012b). This expression pattern mirrored the expression profile of these mucins in human PDAC development (Andrianifahanana et al., 2001; Kaur et al., 2013; Westgaard et al., 2009; Yonezawa et al., 2010). Since the KC model expresses *Kras* constitutively, we were interested in evaluating mucin expression in the inducible model, with a disease course more similar to human PDAC. Similar to the expression profile in KC mice, Muc1 was elevated in the pancreas of iKC mice (starting at week 4 post-tamoxifen) and was expressed at a basal level in the corn-oil-treated control littermates. The pattern of Muc1 expression in iKC and KC models, and in human PDAC tissues (Andrianifahanana et al., 2001; Kaur et al., 2013; Rachagani et al., 2012b; Tsutsumida et al., 2006; Westgaard et al., 2009; Yonezawa et al., 2010) suggests Muc1 is expressed throughout the pancreas irrespective of the cell type from which PDAC originates.

In contrast to Muc1, Muc4 is not expressed in the normal mouse pancreas or in iKC pancreas until 10 weeks post-tamoxifen treatment; but its expression increases progressively up to 50 weeks of treatment. Our findings in the iKC mouse model is in agreement with what we have observed in the KC mouse model (Rachagani et al., 2012b), where *de novo* Muc4 expression occurs by 10 weeks of age and progressively increases as disease severity increases to PDAC. *De novo* expression of Muc5Ac was detected by 40 and 50 weeks post-tamoxifen treatement, with no expression in control animals at any time point. This finding is inconsistent with that observed in the KC model (Rachagani et al., 2012b), where its expression is seen in the pancreas at embryonic day 8.5. This is due to differences between Pdx1 and Ptf1a promoters. Pdx1 Cre-mediated *Kras^G12D^* activation occurs during early embryonic development or in early multipotent progenitors; hence Cre activity is widespread, which causes high levels of Muc5Ac. Conversely, the spatial and temporal Ptf1a-Cre activation of *Kras^G12D^* occurs only during adult developmental stages or in adult progenitor cells. This difference in promoter function might have direct influence on expression of Muc5Ac or other mucin oncoproteins. Overall, the heavily glycosylated transmembrane mucins, Muc4 and Muc16, are similarly expressed in the KC and iKC models.

Previously, we reported that the transcription factor Ncoa3 regulates mucin expression in the pancreas, and is significantly upregulated in the KC model compared to littermate controls (Kumar et al., 2015). Recapitulating this finding, Ncoa3 expression was elevated by 30 weeks post-tamoxifen treatment in the iKC model. In addition, we analyzed the expression profile of FoxM1 and p-cJun transcription factors in the pancreas of the iKC mouse model. After 10 to 50 weeks of tamoxifen injection, nuclear expression of FoxM1 and p-cJun was observed. This studies demonstrating potential binding sites for FoxM1 and c-Jun in the promotors of mucin genes (Joshi et al., 2016; Kong et al., 2013; Singh et al., 2007). Levels of Ncoa3, FoxM1 and p-cJun were increased in conjunction with Muc1, Muc4, Muc5Ac expression, establishing a potential link between these transcription factors and *Kras^G12D^*-regulated mucin expression in PDAC.

Our study characterized *Kras*-mediated mucin (Muc1, Muc4, Muc5Ac and Muc16) expression during PDAC pathogenesis arising from the acinar cell lineage. The findings in the iKC model support our previous findings in the KC model, and further implicate aberrant mucin expression as a mechanism associated with PDAC initiation, progression and aggressiveness. We plan to develop mouse syngeneic cell lines mimicking the biological features of PDAC progression, such as PanIN and PDAC, from the iKC model, as demonstrated previously (Torres et al., 2013). This mouse model and its syngeneic cell lines will be used to test combinations of approved drugs that can potentially improve PDAC treatment. The developed iKC model will also provide a unique opportunity to identify biomarkers relevant to the early onset of PDAC.

## MATERIALS AND METHODS

### Mouse model

Animal procedures were performed in accordance with protocols approved by the University of Nebraska Medical Center Institutional Animal Care and Use Committee, U.S. Public Health Service guidelines, and the International Council for Laboratory Animal Science (ICLAS). The animals involved in this proposed study were treated humanely and in all situations in which the mice are used, pain and discomfort were minimized. B6.129-Krastm4Tyj (01XJ6) mice were obtained from the National Cancer Institute Mouse Models of Human Cancers Consortium (Frederick, MD, USA). The Ptf1a-CreER™ mice were a kind gift from Dr Christopher V. Wright, Vanderbilt University. To generate iKC mice, we crossed *LSLKras^G12D^* mice with *Ptf1aCreER*^TM^ mice to generate *Kras^G12D^;Ptf1a-CreER*™. The resulting F1 progeny were genotyped for *Kras* and *Ptf1a-CreER* by polymerase chain reaction (PCR) using specific primer sets. All positive animals (*Kras^G12D^* and Ptf1a-CreER™) carried the mutant *Kras^G12D^* allele and *Cre* recombinase in the pancreas. To activate the *Kras* (transgenic floxed Kras^G12D^) alleles in the mouse pancreata postnatally (16 weeks after birth, either sex), we intraperitoneally injected iKC mice (three or four mice/group/time point) with tamoxifen (3 mg/mice for 3 alternative days) or corn oil on three consecutive days, which led to excision of the LSL cassette in the tamoxifen group. Corn-oil-injected mice acted as control with an intact LSL cassette in the pancreas. Animals were housed five mice in a group and were kept under a 12 h light/dark cycle at 22±1°C and 50±10% relative humidity with *ad libitum* access to food and water. Animals were euthanized by CO_2_ asphyxiation followed by cervical dislocation at 4, 10, 20, 30, 40 and 50 weeks after tamoxifen/corn-oil injections, and the pancreata were resected and observed for gross metastatic lesions on distant organs. A separate schema demonstrating the design of the study is shown in Fig. S1. A portion of the pancreas tissue was stored in buffered formalin (10%) for IHC analysis. The rest of the pancreas was flash frozen in liquid nitrogen and stored at -80°C for analysis of mucin gene expression (Rachagani et al., 2012b).

### DNA isolation and genotyping

Mouse tails were clipped at 15 days of age and DNA was isolated using the Maxwell^®^ 16 mouse tail DNA purification kit (Promega, Madison, USA) according to the manufacturer's protocol. Genotyping PCR was performed using 100 ng of genomic DNA in 2x PCR master mix (Promega, Madison, WI, USA) and 5 pmol of each forward and reverse primers of *Kras* and *Ptf1a-CreER* genes: forward (*Kras*), 5′-CCT TTA CAA GCG CAC GCA GAC TGT AGA-3′; reverse (*Kras*), 5′-AGC TAG CCA CCA TGG CTT GAG TAA GTC TGC A-3′; forward (*Ptf1a-CreER*), 5′-GTG ATC ATG CAA GCT GGT GGC T-3′; reverse (*Ptf1a-CreER*), 5′-CCG ACT TGA CGT AGC AAG CAA C-3′ in a final volume of 20 μl. PCR conditions consisted of 5 min at 95°C, followed by 40 cycles at 94°C for 1 min, annealing at 59°C for 2 min, followed by extension at 72°C for 45 s. A final extension at 72°C was performed for 10 min. PCR products were analyzed using 1.5% agarose gel electrophoresis (Rachagani et al., 2012b).

### Cell lines

The human PC cell lines SU86.86, AsPC-1, CD18/HPAF, MIA PaCa-2 and HUPT-3 were obtained from ATCC and authenticated by STR profiling method. These PC cell lines were cultured in DMEM supplemented with 10% fetal bovine serum and antibiotics (100 µg/ml of penicillin and streptomycin) at 37°C with 5% CO_2_ in a humidified atmosphere. Stable *Kras^G12D^* allele knockdown PC cell lines were generated by using shRNA specifically targeting the *Kras^G12D^* allele as described previously (Rachagani et al., 2011). The human PC cells lines were also routinely validated for mycoplasma contamination.

### RNA isolation

Total RNA was extracted from CD18/HPAF-Scr and CD18/HPAF-ShKras^G12D^ using the mirVana™ miRNA isolation kit (Applied Biosystems, Austin, TX, USA) according to the manufacturer's instructions. RNA purity and integrity was determined by spectrophotometry (NanoDrop™, NanoDrop Technologies Inc., Wilmington, DE, USA) at λ 260/280 and bioanalyzer microfluid chip assays (Agilent Technologies, Waldbronn, Germany), respectively (Rachagani et al., 2012b).

### RT-qPCR

Total RNA was reverse transcribed into cDNA. Reactions were prepared with 2 μg total RNA, 10 mM dNTPs, and 1 µl oligo dT. Following incubation at 65°C for 5 min, first-strand RT buffer (5×), DTT (0.1 M) and 1 µl RNase inhibitor were added to the above mixture, and reactions were incubated at 42°C for 2 min. To extend the reaction, each reaction tube received SuperScript II RT (50 units) and was incubated at 42°C for 50 min. RT-PCR was carried out on 2 μl cDNA in 1× SYBRGreen PCR Master Mix (2×, Roche Applied Science, Indianapolis, IN, USA) and primers (MUC5AC/β-actin) in a total volume of 10 μl. PCR conditions consisted of 10 min at 95°C, followed by 40 cycles of annealing and extension at 95°C for 15 s and 60°C for 1 min. Transcript copy numbers were normalized to β-actin, which served as an internal control. PCR products were analyzed using 1.5% agarose gel electrophoresis (Lakshmanan et al., 2012, 2016, 2015; Rachagani et al., 2012b).

### Histological analysis

After euthanizing mice at different time intervals (4, 10, 20, 30, 40 and 50 weeks of age), a portion of the pancreas from the F1 progeny of tamoxifen- (floxed Kras^G12D^) and corn-oil- (unfloxed Kras^G12D^) administered animals (*n*=4/group) was fixed in 10% (v/v) buffered formalin (Thermo Fisher Scientific, Fair Lawn, NJ, USA) and embedded in paraffin. Sections of 4 μm thickness were mounted on glass slides, deparaffinized in xylenes, hydrated in a decreasing gradient of ethanol, and stained with H&E as described previously (Rachagani et al., 2012b). The slides were examined under a light microscope (Nikon, Eclipse Ti-S, Japan) (Rachagani et al., 2012b).

### IHC

IHC analysis was performed with the following antibodies: anti-mouse Muc1 (1:200, Clone #HMFG1, Abcam, ab70475), Muc5Ac (1:400, clone 45M1, Abcam), Muc16 (1:10,000, RS synthesis, Louisvile, KY, USA), Ncoa3, pcJun and FoxM1, amylase, CK19 (Santa Cruz Biotechnology, CA, USA) and Muc4 (1:4000, 4A rabbit polyclonal), the latter was designed in our lab and developed by GenScript (Piscataway, NJ, USA). IHC analysis was performed on pancreatic tissue from different age groups as mentioned above. Briefly, after formalin fixation of tissues for 48 h, sections of 4 μm thickness were placed on slides. The slides were incubated overnight at 56°C and then were washed with xylene to remove paraffin. Tissue sections were rehydrated with decreasing concentrations of ethanol, and endogenous peroxidase activity was quenched with 5% H_2_0_2_ in methanol for 1 h. Blocking was performed with 2.5% horse serum for 2 h. Primary antibodies were incubated overnight, and tissue sections were washed three times with phosphate buffered saline (PBS) solution containing 0.05% Tween^®^ 20 (PBST). Anti-mouse universal secondary antibody conjugated with horseradish peroxidase was added for 30 min. Following three PBST washes, mucin antibodies were detected using, 3,3′-diaminobenzidine (DAB, Vector Laboratories, Burlingame, CA, USA) prior to counterstaining with Hemotoxylin. The slides were dehydrated with increasing concentrations of ethanol followed by two xylene washes, and mounted with Permount mounting medium (Fisher Scientific Fair Lawn, NJ, USA). Slides were observed and scored by a certified pathologist. The differential expression of mucins was scored from 0–3, where 0 was considered to be negative; 1, weak; 2, moderate and 3, strong. The percentage of positive cells for each respective antibody was given a score on a scale of 1–4 (where 1, 0-25%; 2, 26–50%; 3, 51–75%; and 4, 76–100%). A composite score (0–12) was calculated by multiplying the value of staining intensity by the value representing percentage of positive cells (Rachagani et al., 2012b).

### Immunofluorescence

Immunofluoresence was performed as described previously (Lakshmanan et al., 2012, 2015; Ponnusamy et al., 2010; Rachagani et al., 2012a).

### Immunoblotting

Immunoblot analysis was performed as described in our previous publications (Lakshmanan et al., 2012, 2015; Ponnusamy et al., 2010; Rachagani et al., 2012a) using the following antibodies: anti-Kras (F234, sc-30) and β-actin (sc-47778) from Santa Cruz Biotechnology MUC5AC (clone CLH2, Novus Biologicals), anti-mouse MUC1 (clone HMFG2), anti-MUC4 (clone 8G7, produced in-house) and anti-MUC16 (clone M11, Dako).

### Statistical analysis

Results are expressed as mean±standard error of mean (s.e.m.). The statistical significance of differences was analyzed by Student's *t*-test to assess staining patterns for each mucin at each interval (different stage) of PC progression.

## Supplementary Material

Supplementary information
