# Supplementary material for: Acinar transformed ductal cells exhibit differential mucin expression in a tamoxifen-induced pancreatic ductal adenocarcinoma mouse model
Source: Biol Open. 2020 Sep 7;9(9):bio052878. doi: 10.1242/bio.052878 (PMC7502593; doi:10.1242/bio.052878)
Supplement: Supplementary information [file biolopen-9-052878-s1.pdf]

Biology Open (2020): doi:10.1242/bio.052878: Supplementary information

Supplementary Figure 1

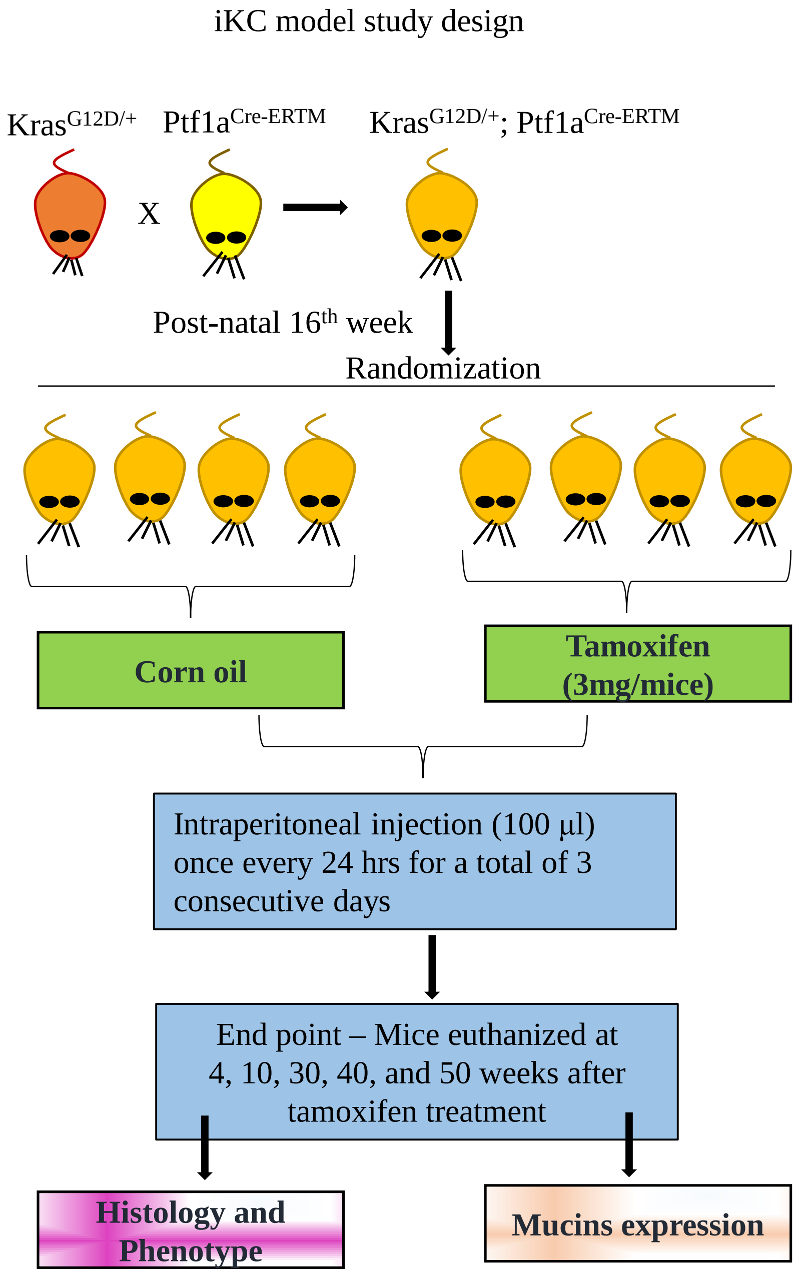

Figure. S1. Flow chart design showing experimental strategies of the iKC mice model. Overview of inducible KC (iKC) model breeding strategy, animal randomization, treatment procedures (route of administration, tamoxifen dosage, schedule), and endpoints were described as a schematic diagram.

Biology Open • Supplementary information

Supplementary Figure 2

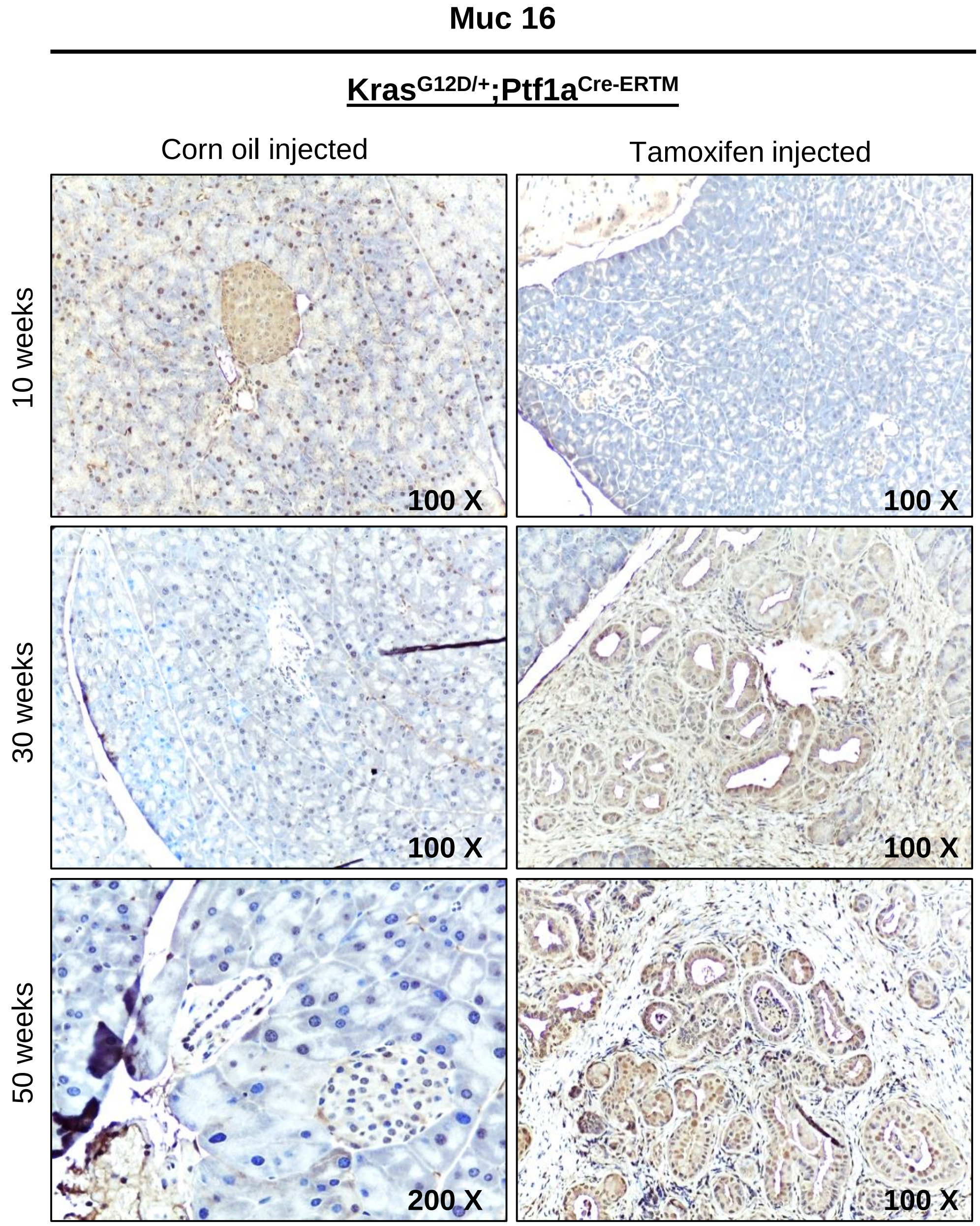

Figure. S2. Expression profile of transmembrane mucin Muc16 in the tamoxifen-induced iKC mouse model. IHC analysis for Muc16 expression was performed on pancreatic tissues isolated from corn oil- and tamoxifen-treated iKC mice. Expression of Muc16 was not detected in corn oil-treated mice (left column) but increased progressively from 10 to 50 weeks following tamoxifen injection (right column).

Biology Open • Supplementary information

Supplementary Figure 3

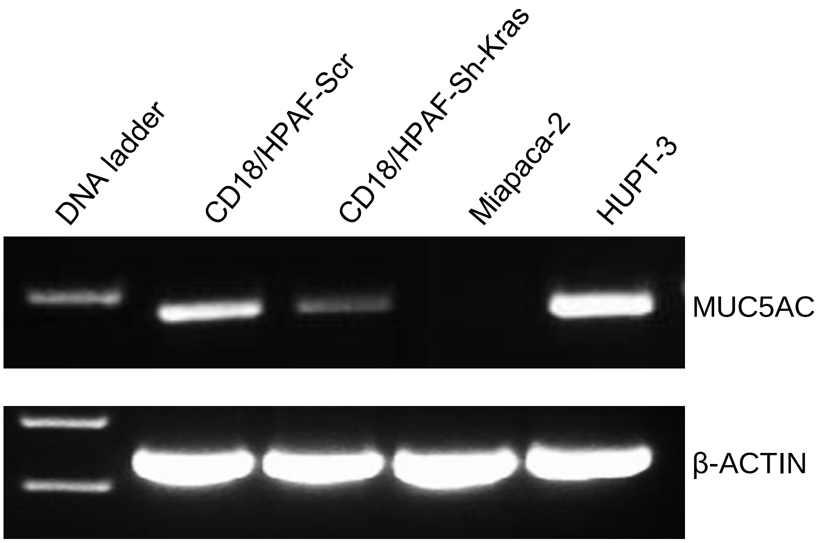

Figure. S3. **Depletion of Kras^{G12D} allele in pancreatic cancer cells.** Knock-down of Kras^{G12D} in CD-18/HPAF using the pRetro.Puro vector carrying an shRNA against mutated Kras led to a significant down-regulation of *MUC5AC*. Beta-actin served as an internal loading control.

Supplementary Figure 4

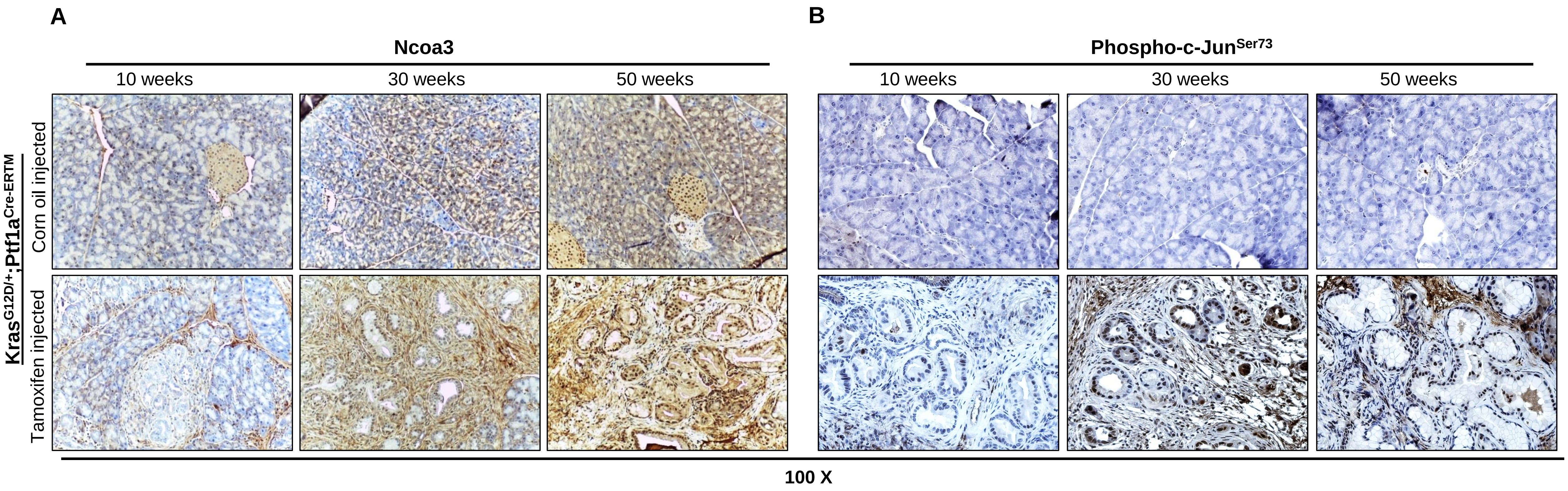

Figure. S4. Expression profile of Ncoa3 and p-cJun in the tamoxifen-induced iKC mouse model. IHC studies using Ncoa3 and p-cJun antibodies were performed on pancreatic tissues isolated from corn oil- and tamoxifen-treated iKC mice. (**A**) Expression of Ncoa3 and (**B**) p-cJun in the nucleus of iKC pancreas cells was low (basal level) but increased upon tamoxifen injection.
